# Electroacupuncture at Zusanli (ST36) Prevents Intestinal Barrier and Remote Organ Dysfunction following Gut Ischemia through Activating the Cholinergic Anti-Inflammatory-Dependent Mechanism

**DOI:** 10.1155/2013/592127

**Published:** 2013-04-04

**Authors:** Sen Hu, Ming-Hua Du, Hong-Min Luo, Huan Wang, Yi Lv, Li Ma, Zhi-Long Lin, Xian Shi, Ingrid Gaischek, Lu Wang, Gerhard Litscher

**Affiliations:** ^1^Research Laboratory of Shock and Multiple Organ Dysfunction, Burns Institute, First Hospital Affiliated to the PLA General Hospital, Beijing 100048, China; ^2^Medical School of Chinese PLA, Beijing 100853, China; ^3^Department of Acupuncture and Moxibustion, Chinese People's Liberation Army General Hospital, No. 28 Fu-Xing Road, Beijing 100853, China; ^4^Stronach Research Unit for Complementary and Integrative Laser Medicine, TCM Research Center Graz, Research Unit of Biomedical Engineering in Anesthesia and Intensive Care Medicine, Medical University of Graz, Auenbruggerplatz 29, 8036 Graz, Austria

## Abstract

This study investigated the protective effect and mechanism of electroacupuncture at ST36 points on the intestinal barrier dysfunction and remote organ injury after intestinal ischemia and reperfusion injury in rats. Rats were subjected to gut ischemia for 30 min, and then received electroacupuncture for 30 min with or without abdominal vagotomy or intraperitoneal administration of cholinergic **α**7 nicotinic acetylcholine receptor (**α**7nAChR) inhibitor. Then we compared its effects with electroacupuncture at nonchannel points, vagal nerve stimulation, or intraperitoneal administration of cholinergic agonist. Cytokine levels in plasma and tissue of intestine, lung, and liver were assessed 60 min after reperfusion. Intestinal barrier injury was detected by histology, gut injury score, the permeability to 4 kDa FITC-dextran, and changes in tight junction protein ZO-1 using immunofluorescence and Western blot. Electroacupuncture significantly lowered the levels of tumor necrosis factor-**α** and interleukin-8 in plasma and organ tissues, decreased intestinal permeability to FITC-dextran, and prevented changes in ZO-1 protein expression and localization. However, abdominal vagotomy or intraperitoneal administration of cholinergic **α**7nAChR inhibitor reversed these effects of electroacupuncture. These findings suggest that electroacupuncture attenuates the systemic inflammatory response through protection of intestinal barrier integrity after intestinal ischemia injury in the presence of an intact vagus nerve.

## 1. Introduction

Intestine ischemia and reperfusion (iI/R) injury are usually secondary to a variety of diseases, such as multiple trauma, severe burn, various forms of shock, intra-abdominal sepsis, surgical operation, and inflammatory bowel disease. Gut I/R injury may result in mucosal epithelial cell damage, loss of basement membrane integrity and barrier function, which promote bacterial translocation and the local production of cytokines. Then bacteria and its endotoxin shift to circulation and remote organs such as lung and liver, contributing to the subsequent local and systemic inflammation, which may lead to systemic inflammation response syndrome (SIRS), multiple organ dysfunction syndrome (MODS), and possibly, death [1, 2]. This evidence suggest that the loss of epithelial cell integrity and barrier function of intestine may very well be a source or promoter for the systemic inflammatory process and MODS. Thus, interventions to prevent the gut epithelial barrier breakdown and to attenuate the local and secondary distant organ inflammatory response would be critical in preventing SIRS or MODS and improving patients' outcome [3, 4]. However, at present, there is still a lack of effective drugs or interventions to protect the gut epithelial barrier. Recent studies showed that electrical stimulation of the vagus nerve could protect intestinal barrier and alleviate inflammatory injury in intestine and remote organs of animals following burn injury by activation of the cholinergic anti-inflammatory pathway [5–8]. However, it is still difficult to apply electrical stimulation to the vagus nerve in clinical practice due to complicated manipulation and untoward side effects, including serious tissue injury.

Acupuncture as one of the therapeutic maneuvers in traditional Chinese medicine (TCM) has been applied in clinics for thousands of years, and it has been found to have a bidirectional neuron-endocrine-immune system regulating effect; also, it antagonizes systemic inflammatory response with no side effects. Studies also showed that acupuncture at Zusanli (ST36) points, which was found to be related to the parasympathetic system, could attenuate inflammatory response and prompt gastrointestinal function [9–11].

Since the effect of acupuncture at ST36 is similar to that of activating the vagus nerve [9, 10, 12], we hypothesize that acupuncture at bilateral ST36 might exert a protective effect on intestinal barrier injury through activation of the vagus nerve pathway and its cholinergic receptor. Therefore, the objectives of our present study are to investigate whether electroacupuncture at ST36 could attenuate the release of tumor necrosis factor-*α*(TNF-*α*) and interleukin-8 (IL-8) in local intestine, alleviate the injury of intestinal villus, reduce the permeability of the intestinal mucosa, regulate the integrity of intestinal tight junctions, prevent degradation of intestinal ZO-1 expression, thus reduce the levels of TNF-*α* and IL-8 in lung, liver, and serum, protect intestinal barrier and remote organ functions. Moreover, we wanted to explore the relationship between electroacupuncture, vagus nerve and *α*7 nicotinic acetylcholine receptor (*α*7nAChR), and the exact mechanism underlying in electroacupuncture ST36 protection of gut barrier and remote organ function.

## 2. Materials and Methods

### 2.1. Animals

Seventy-two male Sprague-Dawley rats, aged 12 weeks, weighing 250 ± 20 g (purchased from the experimental animal farm of Chinese Peking Union Medical College, Beijing), were used for the experiments. Rats were acclimatized for a while in mesh cages in a temperature-controlled room with a 12 h light-dark cycle in the animal quarter of our laboratory and fasted overnight but allowed free access to water until 4 hours before surgery. All research protocols were approved by the Committee of Scientific Research of the First Hospital Affiliated to General Hospital of PLA, China. The experiment was conducted in compliance with the Guide for Care and Use of Laboratory Animals of National Research Council, China.

### 2.2. Intestine Ischemia Model

Rats were anesthetized with 2% isoflurane inhalation (Yeeran Technology Limited, Beijing, China), and the ventral neck and abdomen were shaved and washed with 10% povidone iodine. The rats were secured onto a heating pad to maintain appropriate body temperature during anesthesia. A right cervical neck incision was performed and the right cervical vagus nerve exposed. A 2 cm upper-midline laparotomy incision was performed to identify gastroesophageal junction and expose the dorsal and ventral vagus nerve on the distal esophagus with a Phenix XLT165-LB stereomicroscope (Phenix Optical Instrument Group Company, Jiangxi Province, China). Then, a microvascular clip was placed across the superior mesenteric artery (SMA) for 30 min. After that, the microvascular clip was removed to allow reperfusion for 60 min, and the animals were randomly assigned to various groups [13].

### 2.3. Animal Grouping and Treatments

As illustrated in Table 1, all the animals underwent the same surgical procedure and ischemia-reperfusion injury, and then the rats were randomly assigned to six groups (see Table 1) with 12 rats each and subjected to different treatments. Animals in the ischemia-reperfusion group (IR) underwent electroacupuncture at nonchannel points (SEA) which were located at the bilateral capitulum fibulae apart from outside the fibulae, with the outside condyle center point to about 1 cm place immediately after the ischemia [14]. Those in the electroacupuncture group (EA) underwent electroacupuncture at ST36 points, located at the posterior and lateral side of the knee joint, 5 mm below capitulum fibulae [14], immediately after the ischemia. Those in the vagotomy group (VX) underwent vagotomy of the dorsal and ventral vagus nerve on the distal esophagus prior to electroacupuncture at ST36 points immediately after the ischemia. Those in the *α*-bungarotoxin group (*α*-BGT) underwent intraperitoneal administration of *α*-bungarotoxin (1 *μ*g/kg, Sigma; an antagonist of *α*7 subunit of cholinergic nicotinic receptor) prior to electroacupuncture at ST36 points immediately after the ischemia [15]. Those in the vagus nerve stimulation group (VNS) underwent electrical stimulation at the right cervical vagus nerve, and those in the PNU282987 group (PNU) underwent intraperitoneal administration of PNU282987 (1–10 mg/kg, IP, Sigma; an *α*7nAChR agonist) immediately after the ischemia.

### 2.4. Electroacupuncture at Zusanli (ST36)

Both hind limbs were shaved and the skin was disinfected. ST36 acupuncture point was punctured with a depth of 7 mm, and then the needle was connected with an electroacupuncture apparatus (HANS, LH202H). The stimulation was performed for 30 min using an electric current with the intensity of 2 mA and 2–100 Hz. In the EA group, electroacupuncture at ST36 points was applied immediately after ischemia. In the VX group, surgical abdominal vagotomy was performed immediately prior to reperfusion and then electroacupuncture at ST36 points. In the *α*-BGT group, electroacupuncture was applied immediately after intraperitoneal injection of *α*-bungarotoxin. Rats in the I/R group were subjected to EA at nonchannel points as previously described.

### 2.5. Vagus Nerve Stimulation

Following induction of general anesthesia with inhaled isoflurane and prior to ischemia insult, a right cervical neck incision was performed and the right cervical vagus nerve was exposed. An upper-midline laparotomy incision was performed; the gastroesophageal junction was identified; the dorsal and ventral vagus nerve were visualized; both branches of the vagus nerve were identified and divided on the distal esophagus with a stereomicroscope. Vagal nerve stimulation of the right cervical vagus was performed using a biological function experimental system (BL-420F, Chengdu Taimeng Science and Technology Co., Ltd., China) set at 2 mA, 1 Hz for 20 minutes immediately after ischemia in the VNS group. Rats in the VX group underwent right cervical and upper-midline laparotomy incision and vagotomy of the dorsal and ventral vagus nerve before electroacupuncture at ST36 but did not receive vagal nerve stimulation; all other groups underwent right cervical and upper midline laparotomy incision and exposure of the vagus nerve but did not receive vagal nerve stimulation or vagotomy.

### 2.6. Samples of Blood, Intestine, Lung, and Liver Tissues

Rats were anesthetized with 2% isoflurane inhalation and sacrificed by abdominal aorta exsanguination at 60 min after reperfusion. Systemic blood was drawn by abdominal aorta puncture and placed in heparinized Eppendorf tubes on ice. Plasma was obtained by centrifuging the blood at 10,000 g for 10 minutes at 4°C. Segments of distal small intestine, lung, and liver were harvested and immediately homogenized on ice with a 1 mL denaturing lysis buffer or nondenaturing lysis buffer for Western blot or ELISA. The homogenate was then centrifuged at 10,000 g for 10 min at 4°C. Aliquots of the supernatants of plasma and tissue were stored at −80°C until use. Segments of intestine, lung, and liver were also harvested and snap frozen in liquid nitrogen before storage at −80°C for detection. Segments of intestine were also harvested and fixed in 4% paraformaldehyde for histologic evaluation and immunofluorescence.

### 2.7. Detection of TNF-*α* and IL-8 Levels in Intestine, Lung, Liver, and Plasma

TNF-*α* and IL-8 levels in the plasma, intestine, lung, and liver were assessed using commercially available ELISA kits in accordance with the protocol provided by the manufacturer (Nanjing Jiancheng Corp., China). Supernatants were transferred into fresh tubes for the evaluation. Briefly, after adding 50 *μ*L of assay buffer, 50 *μ*L of samples or standard concentration for TNF-*α* or IL-8 were incubated with 50 *μ*L of diluted Biotin Conjugate for 2 hours at room temperature. After 3 washes, the plates were incubated with Streptavidin-HRP for 1 hour at room temperature. After 3 washes, TMB substrate solution was added to the plates for 15 minutes, and the reaction was stopped with stop solution. The absorbance rate was read at 450 nm. The concentrations of the samples were calculated according to the standard curve. TNF-*α* and IL-8 levels in the plasma were expressed as pg/ml. Intestine, lung, and liver TNF-*α* and IL-8 levels were expressed as picograms per milligram of protein.

### 2.8. Histopathologic Score

Segments of the distal ileum were fixed in 10% buffered formalin, embedded in paraffin, and sectioned. Hematoxylin and eosin staining of the intestine was performed. Sections were viewed via light microscopy and reviewed by a pathologist who was blinded to the experimental groups. Three randomly selected fields from each specimen were graded using a scoring system that characterized gut injury on a scale from 0 to 4 where 0 is normal, no damage; 1 is mild, focal epithelial edema; 2 is moderate, diffuse swelling, and necrosis of the villi; 3 is severe, diffuse pathology of the villi with evidence of neutrophil infiltration in the submucosa; and 4 is major, widespread injury with massive neutrophil infiltration and hemorrhage as previously described [16].

### 2.9. Intestinal Epithelial Permeability

An *in vivo* intestinal permeability assay was performed to assess intestinal barrier function. 60 min after reperfusion, animals were anesthetized with inhaled isoflurane. A midline laparotomy incision was performed, and a 5 cm segment of distal ileum was isolated between silk ties. A solution of 500 *μ*L containing 4-kDa FITC-dextran (25 mg/mL, Sigma, St. Louis, USA) diluted in phosphate-buffered saline (PBS) was injected into the lumen of the isolated segment of intestine. The bowel was returned to the abdominal cavity and the abdomen was closed. Animals were maintained lightly under general anesthesia for 30 minutes, at which time systemic blood was drawn by abdominal aorta puncture and placed in heparinized Eppendorf tubes on ice. Plasma was obtained by centrifuging the blood at 10,000 g for 10 minutes at −4°C. Plasma fluorescence was measured in a fluorescence spectrophotometer (Synergy2; BioTek Multi-Detection Microplate reader, USA) and compared with a standard curve of known concentrations of FITC-dextran diluted in rat plasma.

### 2.10. Immunofluorescence

After deparaffinization, the intestine sections were rehydrated and incubated in citrate buffer (Zhongshan Jinqiao Biotechnology Co., Ltd., Beijing, China) for heat-induced antigen retrieval. After three washes with PBS, sections were incubated with 3% BSA (Zhongshan Jinqiao Biotechnology Co., Ltd., Beijing, China) for 30 minutes to block nonspecific binding sites. The sections were then incubated in the ZO-1 antibody (1 : 100; Life Technologies, Gaithersburg, USA) at 4°C overnight. The following day, after washing with PBS three times, they were treated with Alexa Fluor 488 secondary goat anti-rabbit antibody in 1% BSA for 1 hour at room temperature. Prolong Fade (Antifade Mounting Medium, Beyotime Institute of Biotechnology) was added on placement of cover slips. Images were viewed using the Olympus fluorescence microscope (BX51-DP71) with exposure-matched settings.

### 2.11. ZO-1 Expression

The harvested gut tissues were placed in 1 mL of lysis buffer (50 mM Tris-HCl, PH 7.4; 150 mM NaCl; 1% NP-40; 0.1% SDS), then homogenized and centrifuged at 12,000 g for 10 minutes. Following centrifugation, the supernatant was collected and analyzed for protein concentration. Protein concentrations were determined using a protein assay kit (Applygen Technologies Inc. Beijing, China). Total protein (100 *μ*g) was loaded onto a sodium dodecyl sulfate-polyacrylamide gel (SDS-PAGE gel) and run at 120 volts for 2 hours. After electrophoresis, the protein was transferred to a polyvinylidene difluoride membrane (PVDF; Applygen Technologies Inc. Beijing, China) and blocked for 2 hours in TBST (50 mM Tris; 150 mM NaCl; 0.05% Tween 20) containing 5% milk (Applygen Technologies Inc. Beijing, China). The membrane was then incubated with the primary antibodies against GAPDH (1 : 5000; Zhongshan Jinqiao Biotechnology Co., Ltd., Beijing, China), and ZO-1 (1 : 500; Life Technologies, Gaithersburg, USA) at 4°C overnight. After 3 washes in TBST, the membrane was then incubated with corresponding secondary antibodies conjugated to horseradish peroxidase at room temperature for 30 minutes, and chemiluminescence detection was performed by using SuperECL Plus (Applygen Technologies Inc. Beijing, China). Films were developed using a standard photographic procedure. Quantitative analysis of detected bands was carried out by densitometer scanning (ImageJ).

### 2.12. Statistical Analysis

SPSS 13.0 statistical software was used, and all results were expressed as mean ± standard error of the mean. One-way ANOVA was used for comparison among all groups, followed by the Student-Newman-Keuls (SNK) test for comparison between two groups. Differences were considered to be statistically significant, when *P* ≤ 0.05.

## 3. Results

### 3.1. Electroacupuncture at ST36-Lowered TNF-*α* and IL-8 Levels of Intestine, Lung, Liver, and Plasma

Tables 2 and 3 illustrate the effect of electroacupuncture at ST36 on TNF-*α* and IL-8 levels of intestine, lung, liver, and plasma. Intestinal I/R injury causes an inflammatory response in local intestine and remote organs. The levels of TNF-*α* in plasma and tissues of intestine, lung, and liver in the EA group were significantly lower than those in the IR group 60 min after I/R (*P* < 0.05), while the levels of TNF-*α* in animals that received electroacupuncture after vagotomy or *α*-BGT injection were not statistically different from those in the IR group. The TNF-*α* in VNS and PNU groups were obviously lower than those in the IR group (*P* < 0.05). Similarly, the levels of IL-8 in plasma and tissues of intestine, lung, and liver in the EA group were significantly lower than those in the IR group (*P* < 0.05). The elevation of IL-8 concentrations was significantly inhibited in rats having received vagus nerve stimulation and PNU282987 administration compared with that of IR group (*P* < 0.05). However, there was no statistically significant difference in the levels of IL-8 among the VX, *α*-BGT, and IR group.

### 3.2. Electroacupuncture at ST36 Prevented Intestinal Injury

**Figure 1:**
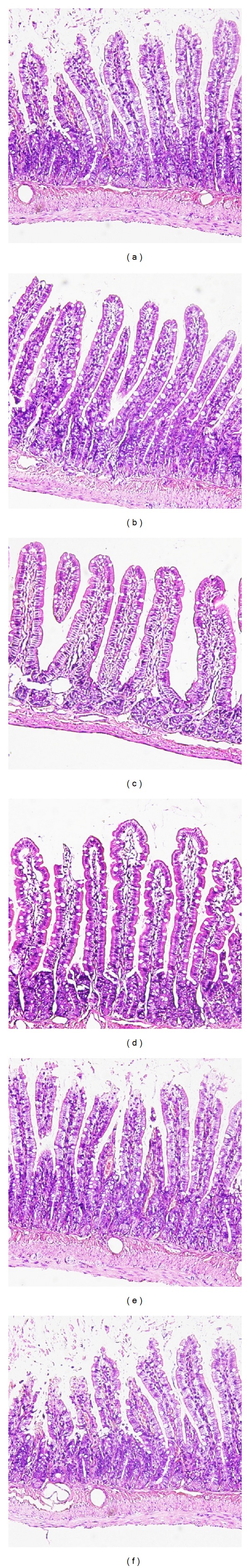
Intestinal histology at 60 min after reperfusion. Electroacupuncture at ST36 protects against intestinal injury after I/R equally as VNS and PNU282987; whereas electroacupuncture at nonchannel points and electroacupuncture at ST36 after abdominal vagotomy or administration of *α*-BGT eliminates such protection. Sections of the distal ileum were harvested 60 min after reperfusion (*n* is 12 animals per group) and stained with hematoxylin and eosin. All images are taken at ×200 magnification with black bar = 5 *μ*m. (a) animals in IR group, (b) animals in EA group, (c) animals in VNS group, (d) animals in PNU group, (e) animals in VX group, and (f) animals in *α*-BGT group.

Sections of the distal ileum from animals in the IR group were compared with those that underwent electroacupuncture at ST36 with or without abdominal vagotomy, administration of *α*-BGT, vagal nerve stimulation, and administration of PNU282987.  Figure 1(a) demonstrates the histologic pattern observed in animals receiving only electroacupuncture at nonchannel points after ischemia with villous tip necrosis, blunting, and sloughing of villi. When electroacupuncture at ST36 was performed immediately after ischemia, there was minimal if any evidence of histologic injury (Figure 1(b)). The same normal pattern of hematoxylin and eosin staining was observed in animals that underwent VNS or PNU282987 administration immediately after ischemia (Figures 1(c), and 1(d)). In contrast, when abdominal vagotomy or administration of *α*-BGT was performed and as such, the cholinergic anti-inflammatory neuroenteric axis was interrupted, electroacupuncture at ST36 failed to prevent the histologic changes induced by I/R injury in the gut (Figures 1(e), and 1(f)). Taken together, these data demonstrated that an intact vagus nerve was necessary for the biological effect of electroacupuncture at ST36 and *α*7nAChR is involved in this effect.

**Figure 2:**
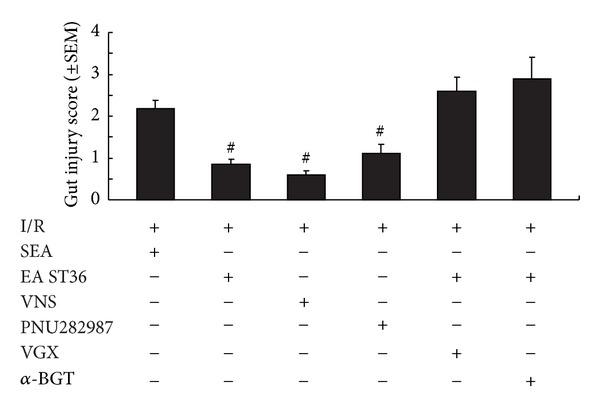
Gut injury scores at 60 min after reperfusion. Gut injury was scored by a pathologist blinded to the experimental groups on a scale of 0 to 4, from no injury to major, widespread injury with a massive inflammatory cell infiltration (Section 2).^#^ versus IR group, *P* < 0.05 (*n* = 12 animals per group).

Gut injury scores were all increased in all six groups due to I/R injury (see Figure 2). Animals subjected to I/R injury with electroacupuncture at non-channel points had an average injury score that was significantly higher than that of EA, VNS, and PNU282987 groups (2.18 ± 0.19 versus 0.85 ± 0.13, 0.6 ± 0.09, 1.1 ± 0.23; *P* < 0.05, resp.). Surgical abdominal vagotomy or administration of *α*-BGT before electroacupuncture at ST36 eliminated the protective effects with results similar to animals subjected to I/R injury with electroacupuncture at non-channel points (2.6 ± 0.33, 2.9 ± 0.19 versus 2.18 ± 0.19; *P* < 0.05, resp.).

### 3.3. Electroacupuncture at ST36 Lowered Intestinal Permeability

**Figure 3:**
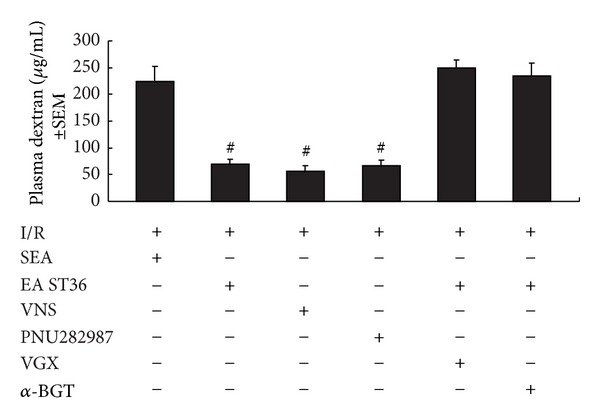
Intestinal permeability to 4-kDa FITC-dextran at 60 min after gut I/R injury. Electroacupuncture at ST36, VNS, and PNU282987 similarly protected the intestine from an increase in permeability after I/R injury, whereas abdominal vagotomy before EA ST36 eliminates such protection. The administration of *α*-BGT before EA ST36 also eliminates such protection. ^#^ versus IR group, *P* < 0.05 (*n* = 12 animals per group).

Animals in the IR group had an increase in permeability, when compared with sham animals (data not shown). Electroacupuncture at ST36, VNS, and administration of PNU282987 all protected against increased intestinal permeability compared with IR group receiving electroacupuncture at non-channel points (69.65 ng/mL ± 8.65 ng/mL, 56.37 ng/mL ± 10.95 ng/mL, and 66.84 ng/mL ± 11.10 ng/mL versus 225.36 ng/mL ± 28.12 ng/mL). However, when abdominal vagotomy or administration of *α*-BGT was performed before reperfusion, the intestinal permeability of animals undergoing electroacupuncture at ST36 was indistinguishable from electroacupuncture at non-channel points (249.67 ng/mL ± 14.67 ng/mL and 234.5 ng/mL ± 23.5 ng/mL versus 225.36 ng/mL ± 28.12 ng/mL) (see Figure 3). These data indicated that electroacupuncture at ST36 can only offer protection to the gut in the presence of intact neuroenteric innervation, and electroacupuncture at ST36 might exert this effect via *α*7nAChR.

### 3.4. Electroacupuncture at ST36 Preserves the Integrity of Intestinal Tight Junctions and Prevents Degradation of Intestinal Z0-1 Expression

**Figure 4:**
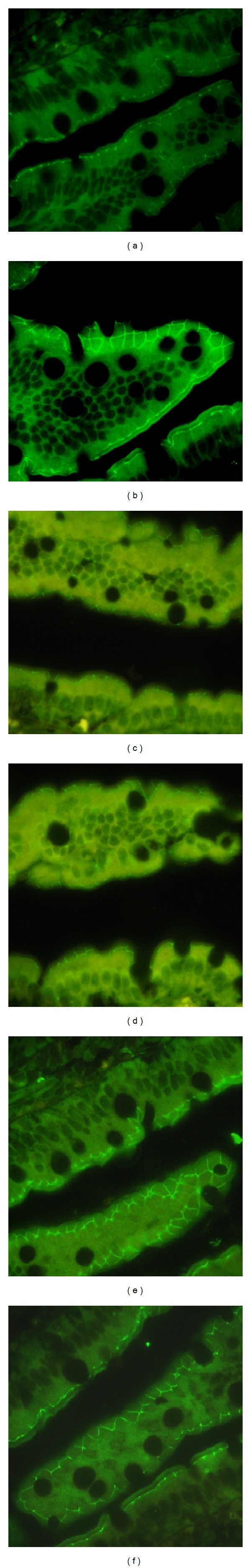
Intestinal ZO-1 immunofluorescence at 60 min after reperfusion. Animals in the IR group showed a low fluorescent intensity at the cell periphery after iI/R injury (a), and EA ST36 showed preservation of the robust structure of ZO-1 staining (b), VNS and PNU282987 similarly prevented the degeneration of ZO-1 (c and d), abdominal vagotomy or *α*-BGT administration performed before EA ST36 eliminates the protective effect (e and f). All images are taken at ×400 magnification with black bar = 5 *μ*m (*n* = 12 animals per group, size bar = 2 *μ*m).

It has been found that tight junction proteins are critical structural proteins in the maintenance of mucosal barrier function [17, 18]. We used immunofluorescent staining of the tight junction protein, ZO-1, to assess the integrity of intestinal tight junctions. The tight junction protein, ZO-1, undergoes protein expression alterations in response to I/R injury. Exposure-matched fluorescent intensity correlated to the amount of ZO-1 protein expression after immunostaining (Figure 4). After I/R injury, animals treated with electroacupuncture at nonchannel points showed a reduction in ZO-1 expression evidenced by a low fluorescent intensity at the cell periphery (Figure 4(a)). Animals treated with electroacupuncture at ST36 immediately after ischemia (Figure 4(b)) showed preservation of the robust structure of ZO-1. In contrast, in animals treated with electroacupuncture at ST36 after surgical abdominal vagotomy or administration of *α*-BGT, no protection was afforded to the intestinal mucosa evidenced by the ready interruption and partial disappearance of ZO-1 staining at the cell periphery in villous epithelial cells (Figures 4(c) and 4(d)). Vagal nerve stimulation or IP PNU282987-treated animals had ZO-1 expression similar to EA group evidenced by a high fluorescent intensity and intense localization of ZO-1 staining at villous enterocytes (Figures 4(e) and 4(f)).

**Figure 5:**
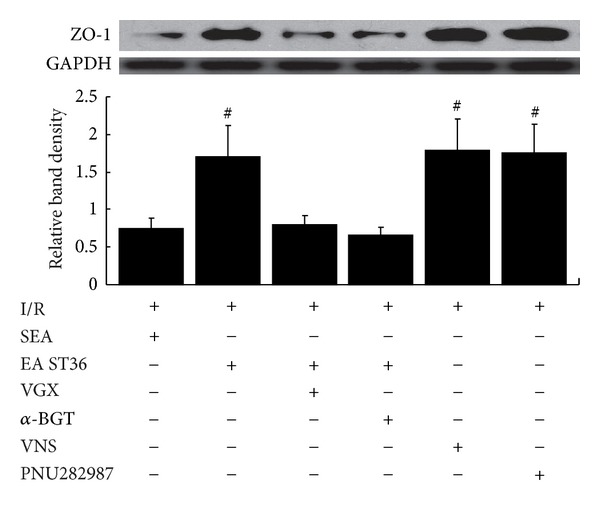
Electroacupuncture at ST36 prevents the decrease of intestinal ZO-1 protein expression. Intestinal extracts were obtained from animals 60 min following reperfusion for measurement of ZO-1 protein expression using Western blot. Representative Western blot for the ZO-1 protein is shown with its corresponding GAPDH loading control to demonstrate equal protein load in all lanes. EA ST36, VNS, and PNU282987 resulted in preservation of protein expression when performed on animals with an intact neuroenteric axis. Significant reduction in ZO-1 expression was seen after iI/R treated with SEA, and if abdominal vagotomy or *α*-BGT was performed before EA ST36. ^#^ versus IR group, *P* < 0.05 (*n* = 12 animals per group).

These results were confirmed by Western blotting for the ZO-1 protein in intestinal tissue lysates (Figure 5). When compared with the average relative band density of animals treated with electroacupuncture at nonchannel points after ischemia, animals treated with electroacupuncture at ST36 had significantly higher ZO-1 expression (*P* < 0.05). In contrast, intestinal ZO-1 protein levels were significantly decreased in animals that underwent surgical abdominal vagotomy or administration of *α*-BGT before electroacupuncture at ST36. Similar to electroacupuncture at ST36, vagal nerve stimulation or administration of PNU282987 after ischemia maintained a significantly higher level of ZO-1 expression compared with animals in IR group (*P* < 0.05).

## 4. Discussion

Recently, researchers have demonstrated an expanded role for vagus nerve stimulation and the parasympathetic anti-inflammatory mechanism that provides a local protective effect on the gut against epithelial barrier dysfunction [5–7]. The difficulty in translating these findings to clinical implications is that direct electrical stimulation of the vagus nerve is impractical in acutely injured patients for its complicated operation and adverse effects. Therefore, a more clinically desirable alternative therapy needs to be established, ideally an approach that has similar effects as vagus nerve stimulation and can be applied during the resuscitative phase of trauma care. In this research, the actions of electroacupuncture (EA) at ST36 acupoints in rats demonstrated protective effects on reducing local gut inflammation and intestinal barrier breakdown through activating the cholinergic anti-inflammatory-dependent mechanism and involved *α*7nAChR. To our knowledge, this is the first time that ZO-1 has been used in evaluating the effect of EA in preventing intestinal barrier breakdown.

In traditional Chinese medicine (TCM), an overall health is viewed as the maintenance of dynamical balance of Yin and Yang, and their imbalance can result in the development of diseases [19]. The balance of Yin and Yang is regulated by the smooth flow of “Qi”, a concept referred to as a vital force or energy in TCM that circulates between the organs along meridians. EA is one of the mostly used therapies to balance Yin and Yang by improving the flow of Qi along the meridians through stimulation of acupoints. EA is a modification of conventional acupuncture that stimulates acupoints with electrical current instead of manual manipulations and appears to have more consistently reproducible results in both clinical and research settings. EA has been clinically used to prevent and treat gastrointestinal diseases for years and can accomplish satisfying results. ST36 is a specific acupoint located on the foot Yang Ming stomach meridian and the “lower He-sea point”, which means the Qi of this meridian connects with this point, to this channel. This acupoint is known to strengthen the Qi, not only the stomach Qi, even though this acupoint belongs to the stomach meridian but also the general Qi in the whole body. ST36 is one of the most frequently used acupoints that can be stimulated through needles or “moxibustion” to balance and harmonize Yin and Yang by improving the flow of Qi along the meridians. For this reason, ST36 is the target to treat various diseases in the gastrointestinal tract as well as general symptoms in the whole body [20]. That may be an explanation of why stimulation at ST 36 points attenuated the inflammatory action in liver and lung in this research.

Previous clinical studies have proved that acupuncture can positively affect gastrointestinal tract disorders, such as stomachache, abdominal pain and distension, constipation, diarrhea, vomiting, dysentery, indigestion, and others [21]. Animal experimental studies have showed effects of acupuncture on gastrointestinal motor dysfunction, visceral pain, gastrointestinal secretion and sensation, and gastric and intestinal motilities [22–28]. These effects seem to be relevant to vagus nerve. ST36 acupuncture has also been shown to activate the parasympathetic efferent pathway [9]. For instance, EA at ST36 promoted the gastric myoelectric activity, which was regulated by the vagus, and substance P in the dorsal vagal complex may be involved in the excitatory effects. After bilateral vagotomy, the excitatory effect was completely abolished, suggesting that it was mediated by the vagus [10]. More interestingly, increasing data indicate that stimulation at ST36 points can exhibit significant anti-inflammatory effects. ST36 acupuncture has been shown to inhibit TNF-*α* production [29], attenuate trauma-induced immunosuppression [30], and reverse sepsis-induced neutrophil igration impairment in septic rats [31]. EA at ST36 has also been proved to reduce inflammation in different animal models such as arthritic mice [32] and peritonitis [33]. Collectively, these data suggest the therapeutic potential of acupuncture stimulation of ST36 against gastrointestinal disorders through activation of the parasympathetic efferent pathway.

Judging from these data, we speculate that ST36 acupuncture may partly act through activating the cholinergic anti-inflammatory pathway and, most probably, through the *α*7 subunit of nicotinic receptor to exert its anti-inflammatory effects. Because of its success in reducing inflammation, we sought to apply EA to a rat intestine ischemia and reperfusion model and determine its efficacy in reducing local gut inflammation and intestinal barrier breakdown. To our knowledge, this is the first time that ZO-1 has been used in evaluating the effect of EA in preventing intestinal barrier breakdown.

In this set of experiments, we stimulated ST36 points during ischemia, established its efficacy, and compared its effects with direct VNS and PNU282987 administering. We demonstrated that EA at ST36 points is as effective as VNS in preventing intestinal barrier breakdown after an ischemia and reperfusion insult. Furthermore, we have shown, as a proof of concept, that its biological effect is dependent on an intact vagus nerve and perhaps involves *α*7nAChR.

In our previous studies, we did a lot of work on the effects of EA at ST36 and also proved that it can alleviate intestinal proinflammatory factors, tissue edema, and insult of intestinal mucosa [34], significantly protect tumor necrosis factor-*α* induced-multiple organ dysfunction in rats with sepsis [35], and have significant effects on promoting gastric emptying in rats with 40% blood volume loss [36]. Data from this study also demonstrated that ST36 acupuncture significantly attenuated the expression of cytokine in both local and remote tissues. These data suggest the anti-inflammatory potential of the use of ST36 acupuncture against intestine ischemia and reperfusion insult. Ischemia and reperfusion insult can lead to increased local production of cytokines and further induce entry of these products into the systemic circulation and distant organs. Electrical stimulation of ST36 in rats with ischemia and reperfusion injury significantly blunted local gut cytokine levels, reduced the circulating serum levels of TNF-*α* and IL-8, decreased distant lung and liver levels of TNF-*α* and IL-8, which conduce to prevent SIRS or MODS. Sham acupuncturing or the vagotomy groups increased the production of TNF-*α* and IL-8, which suggested that the anti-inflammatory effect of EA may arise through the vagal nerves, and their integrity was essential.

EA at ST36 points effectively prevented histologic injury of the gut mucosa and maintained intestinal tight junction protein expression and function. Its protective effects were eliminated by disrupting the neuroenteric axis via surgical abdominal vagotomy or partly reversed by *α*7nAChR antagonist *α*-bungarotoxin.

In these experiments, EA at ST36 points successfully maintained low gut injury scores after intestine ischemia and reperfusion, reduced permeability of the distal ileum to 4-kDa FITC-dextran, and maintained normal expression of the tight junction protein ZO-1. We also proved that this biological effect is dependent on an intact vagus nerve. Disrupting the neuroenteric axis via surgical abdominal vagotomy abolished any protective effect of EA at ST36 points.

In summary, data from this study would demonstrate that, for the first time, EA at ST36 points located on a meridian, had protection effects against intestinal barrier dysfunction similar to that seen after VNS or administration of *α*7nAChR agonist. These preclinical animal studies demonstrate that in addition to the use of specific cholinergic agonists or vagus nerve stimulation, EA could be a potential therapeutic asset in the treatment of the severely injured patients with inflammatory diseases. Moreover, it demonstrated that a noninvasive method of transcutaneous EA at ST36 points, which has shown to limit inflammatory responses and improve outcomes, is feasible in clinic and can have major clinical implications. We think that this new data presented here should lead to further study focusing on the effects of EA at ST36 points.

## Figures and Tables

**Table 1:** Groups and treatments.

Groups	Treatments						
	I/R	SEA	EA	Vagus nerve stimulation	PNU282987	Vagotomy	*α*-BGT
IR	+	+	−	−	−	−	−
EA	+	−	+	−	−	−	−
VNS	+	−	−	+	−	−	−
PNU	+	−	−	−	+	−	−
VX	+	−	+	−	−	+	−
*α*-BGT	+	−	+	−	−	−	+

Groups: IR, electroacupuncture at nonchannel points (SEA); EA, electroacupuncture at ST36 acupoints; VNS, vagus nerve stimulation; PNU, PNU282987; VX, vagotomy and electroacupuncture at ST36 points; *α*-BGT, *α*-bungarotoxin and electroacupuncture at ST36 points.

**Table 2:** The effects of EA at ST36 on the levels of TNF-*α* in plasma, intestine, lung, and liver in rats with iI/R injury (mean ± SD, *n* = 12).

Groups	TNF-*α*			
	Intestine	Lung	Liver	Plasma
IR	8.4 ± 1.1	6.6 ± 0.85	4.3 ± 0.79	18.63 ± 3.1
EA	3.9 ± 0.65^#^	2.5 ± 0.5^#^	1.1 ± 0.4^#^	12.3 ± 1.1^#^
VNS	3.1 ± 0.6^#^	2.4 ± 0.7^#^	1.4 ± 0.35^#^	9.2 ± 2.9^#^
PNU	3.5 ± 0.5^#^	2.3 ± 0.51^#^	1.5 ± 0.5^#^	13.55 ± 2.0^#^
VX	8.7 ± 1.0	6.5 ± 0.74	3.9 ± 0.97	24.3 ± 3.8
*α*-BGT	8.2 ± 1.13	6.8 ± 0.62	4.4 ± 0.65	22.13 ± 4.2

The content of TNF-*α* in plasma was expressed as ng/mL, and in intestine, lung, and liver expressed as pg/mg protein in rats. Number versus IR group, *P* < 0.05.

**Table 3:** The effects of EA at ST36 on the levels of IL-8 in plasma, intestine, lung, and liver in rats with iI/R injury (mean ± SD, *n* = 12).

Groups	IL-8			
	Intestine	Lung	Liver	Plasma
IR	13.62 ± 3.08	7.70 ± 1.55	5.93 ± 0.91	24.02 ± 5.53
EA	5.41 ± 1.71^#^	3.68 ± 0.96^#^	3.34 ± 0.56^#^	10.43 ± 3.04^#^
VNS	4.02 ± 0.88^#^	3.53 ± 0.52^#^	3.24 ± 0.53^#^	9.78 ± 2.06^#^
PNU	5.22 ± 1.64^#^	4.38 ± 1.5^#^	3.44 ± 0.39^#^	13.11 ± 5.09^#^
VX	13.76 ± 2.93	7.56 ± 1.5	5.14 ± 1.13	36.24 ± 4.36^#^
*α*-BGT	13.52 ± 2.63	8.0 ± 1.5	6.12 ± 0.75	31.54 ± 6.58^#^

The content of IL-8 in plasma was expressed as ng/mL, and in intestine, lung, and liver expressed as pg/mg protein in rats. ^#^versus IR group, *P* < 0.05.
